# 

**Published:** 1919-11-22

**Authors:** 


### THE HOSPITAL

**The Modern Newspaper of Administrative Medicine and Institutional Life.**

Telegraphic Address: OSPEDALE, RAND, LONDON. Telephone Number: 2734 GERRARD

No. 1746, Vol. LXVII. | Saturday, November 22nd, 1919. | PRICE TWOPENCE

